# Effects of light intensity on growth and leaf functional trait plasticity of *Paris polyphylla var. yunnanensis*

**DOI:** 10.1080/15592324.2026.2709892

**Published:** 2026-08-07

**Authors:** Guicai Zi, Qinghua Li, Yingtian Leng, Jiamo Hu

**Affiliations:** a Lijiang Forestry Research Institute, Lijiang, Yunnan, China

**Keywords:** *Paris polyphylla var. yunnanensis*, light intensity, light signaling, leaf functional traits, photosynthetic physiology, biomass allocation, shade adaptation

## Abstract

To elucidate the light adaptation mechanism and optimal cultivation light regime for *Paris polyphylla var. yunnanensis*, a two-year continuous field positioning trial was conducted with five light gradients (20%, 40%, 60%, 80%, and 100% natural light, PAR = 300–2200 μmol·m^−2^·s^−1^). We systematically investigated the regulatory effects of light intensity on leaf phenotypic traits, anatomical and physiological functions, photosynthetic performance, vegetative growth and rhizome biomass accumulation. Significant differences in plant growth, leaf morphology and all functional trait indices were detected among the treatments (*p* < 0.05). The plants cultivated under 40%–60% natural light (PAR = 700–1400 μmol·m^−2^·s^−1^) exhibited balanced leaf anatomy, maximum photosynthetic efficiency and the highest rhizome fresh biomass. Compared with CK (100% natural light), plants under 40%–60% natural light showed 164.8% greater plant height, 35.5% thicker stem diameter, 50.0% larger rhizome diameter and 124.4% higher rhizome fresh weight. In contrast, 20% natural light induced excessive elongation, slender stems and underdeveloped mechanical tissues, with rhizome fresh weight, length and diameter decreasing by 52.2%, 61.4%, and 62.1%, respectively, relative to CK. Treatments of 80%–100% natural light triggered severe high-light stress, causing leaf scorch, chlorosis and suppressed stomatal development, with most assimilates allocated to antioxidant defense and thermal dissipation rather than rhizome carbon partitioning. In conclusion, *P. polyphylla var. yunnanensis* exhibits a clear light adaptation threshold with distinct gradient-specific responses: 80%–100% natural light induces photoinhibition and suppresses stomatal development; 20% natural light causes excessive elongation, slender stems and reduced rhizome biomass; while 40%–60% natural light optimizes leaf morphology, photosynthetic physiology and biomass allocation, resulting in the highest rhizome fresh weight (63.54–64.4  g) and overall growth performance. The 40%–60% natural light regime (PAR 700–1400 μmol·m^−2^·s^−1^) coordinates leaf morphogenesis, photosynthetic carbon fixation and dry matter accumulation, and is therefore identified as the optimal light condition for high-yield and high-quality medicinal production. This study provides quantitative light regulation parameters (PAR thresholds and corresponding growth responses) for understory bionic cultivation and precise light environment management in facility cultivation of this medicinal herb.

## Introduction

*Paris polyphylla var. yunnanensis* is a perennial herbaceous medicinal species of Melanthiaceae endemic to Southwest China. Its rhizome is rich in steroidal saponins (polyphyllins I, II, VI, and VII) with prominent anti-tumor, anti-inflammatory, immunoregulatory, and hemostatic bioactivities, so this species has long been officially recorded in the Chinese Pharmacopoeia.^1^ Long-term over-harvesting of wild populations and natural habitat degradation have drastically reduced the number of wild resources, and this species has been listed as a National Class II Key Protected Wild Plant of China. Standardized artificial cultivation has become an essential pathway to relieve wild resource shortages and realize sustainable utilization of medicinal germplasm.^2^ As an obligate shade-tolerant medicinal herb, *P. polyphylla var. yunnanensis* has strict environmental requirements during growth and development; suitable light, temperature, air humidity and loose fertile soil are prerequisites for healthy growth.^3^ ^,^ ^4^ Current artificial cultivation modes are divided into understory bionic cultivation and greenhouse facility cultivation. Understory bionic cultivation simulates native understory scattered light habitats, and the total saponin content of rhizomes is 12%–18% higher than that of greenhouse-cultivated plants, which facilitates high medicinal quality formation. However, this cultivation mode is constrained by uncontrollable forest canopy density, litter decomposition and a dynamic understory microclimate, leading to unstable plant growth performance.^5^ ^,^ ^6^ Greenhouse facility cultivation enables artificial environmental regulation but requires high investment and complicated field management; secondary soil salinization and phytotoxicity frequently occur under enclosed cultivation, limiting medicinal yield and quality.^7^ High-quality cultivation of *P. polyphylla var. yunnanensis* must be fully integrate its native ecological characteristics, comprehensively optimize core environmental factors (light, soil, climate), germplasm breeding and agronomic management, and regulate rhizosphere microhabitats. Construction of a four-dimensional coupled cultivation system covering “environment, soil, germplasm, and management” can synergistically improve the seedling survival rate, growth vigor, rhizome yield and medicinal quality.^8^ ^,^ ^9^ Among all environmental factors, light acts as a core upstream signal regulating plant photosynthesis, leaf morphogenesis and secondary metabolite biosynthesis and it determines the growth, development and medicinal quality formation of *P. polyphylla var. yunnanensis.* ^10^ ^,^ ^11^ In natural wild habitats, *P. polyphylla var. yunnanensis* distributes in understory scattered light environments with 2000–8000 lx light intensity, with a narrow light adaptation range. Direct intense full sunlight causes leaf scorch and suppresses photosynthetic efficiency; light intensity below 1500 lx induces excessive internode elongation, slender stems and insufficient biomass accumulation. Meanwhile, extreme low light restricts saponin biosynthesis and accumulation, drastically reducing medicinal yield and quality.^12^ ^,^ ^13^ Previous studies have confirmed that under low-light conditions, *P. polyphylla var. yunnanensis* enhances light interception capacity via coordinated plastic adjustment of leaf morphology and anatomical structures; moderate shading optimizes leaf nitrogen allocation and maximizes key growth indices, including plant height, rhizome dry weight and tiller number.^14^ ^,^ ^15^ Existing research on the light responses of *P. polyphylla var. yunnanensis* mostly relies on single-growing-season static observation, lacking two-year continuous comparative analysis of interannual adaptive differences under multi-gradient light treatments. The coupling relationship between leaf functional trait plasticity and rhizome active medicinal component accumulation remains unclear, and growth response rules under multi-factor field interaction have not been fully clarified.^16^ Furthermore, the light adaptation threshold, dynamic light signal response characteristics, and quantitative relationships between core phenotypic traits (leaf area, specific leaf area, and stomatal density) and light intensity are poorly understood. Systematic experimental verification of coordinated morphological, anatomical and physiological responses under gradient light is still absent.^17^ ^,^ ^18^

Based on the main production area conditions in Lijiang, Yunnan Province, we set five continuous light intensity gradients (20%–100% natural light, PAR = 300–2200 μmol·m^−2^·s^−1^) and carried out a two-year consecutive field controlled-lightfield-controlled light positioning experiment. The objectives of this study were: (1) to systematically explore the effects of light intensity on the leaf phenotypic traits, leaf anatomical construction, vegetative growth and photosynthetic physiological performance of *P. polyphylla var. yunnanensis*; (2) to clarify the suitable light ecological range for healthy growth and high-yield cultivation; (3) to reveal the adaptive mechanisms of leaf function and photosynthetic physiology responding to gradient light signals; and (4) to provide a theoretical basis and quantitative technical parameters for optimized shading regulation and standardized high-quality cultivation of this valuable medicinal herb.

## Materials and methods

### Experimental design

The two-year field positioning experiment was conducted from January 2024 to October 2025. A completely randomized block design was adopted, with five gradient light intensity treatments established: 100% natural light (designated as CK), 80% natural light, 60% natural light, 40% natural light, and 20% natural light. Each light treatment contained six independent biological replicate plots, with 24 uniform seedlings planted per replicate plot, forming a total of 720 experimental individuals. Uniform planting spacing of 30 cm × 30 cm was implemented across all the plots. Height-adjustable light isolation baffles (1.2 m) were installed between adjacent blocks to eliminate cross-interference of scattered light and guarantee independent light environments for each treatment.

Gradient light environments were constructed using black shade nets with five different light transmittance levels. The five light intensity treatments were achieved by covering the experimental plots with black shade nets of different densities (2-needle, 3-needle, 4-needle, 5-needle, and 6-needle, respectively). The shade nets were suspended 1.2 m above ground level using height-adjustable frames. The light transmission rates of the five shade nets were verified using a LI-1500 quantum sensor prior to installation. The detailed light regime and corresponding photosynthetically active radiation (PAR) range for each treatment are listed below:

100% natural light (CK): PAR = 1800–2200 μmol·m^−2^·s^−1^

80% natural light: PAR = 1400–1800 μmol·m^−2^·s^−1^

60% natural light: PAR = 1000–1400 μmol·m^−2^·s^−1^

40% natural light: PAR = 700–1000 μmol·m^−2^·s^−1^

20% natural light: PAR = 300–600 μmol·m^−2^·s^−1^

Continuous dynamic light monitoring was performed using LI-1500 quantum sensors throughout the experimental period. Three uniform monitoring points were arranged in each replicate plot, and PAR was recorded weekly from 10:00–11:00 AM to ensure the consistency of the light treatments. The height of the shade nets was adjusted monthly to maintain stable PAR gradients and guarantee data accuracy and repeatability.

All non-light environmental variables were strictly homogenized across the treatments as controlled variables: an integrated water–fertilizer drip irrigation system was used to supply synchronous quantitative water and nutrients; unified ventilation, humidity reduction and pest control management were implemented to eliminate interference from soil moisture, nutrient difference, and biotic stress. Light intensity was set as the sole experimental variable to ensure the rigor of the controlled experiment.

### Plant materials and site environmental conditions

#### Experimental site

The field trial was carried out at the medicinal planting base of Yulong Qiushi Agricultural Development Co., Ltd., Jiazi Village, Daju Town, Yulong County, Lijiang City, Yunnan Province (100°16′15″ E, 27°11′50″ N). The experimental site is at an altitude of 2400 m with a field slope of 15–25°, representing typical mountainous cultivation habitats for wild and artificially cultivated *P. polyphylla var. yunnanensis*. The regional climatic conditions: annual average temperature = 10°C, annual precipitation ≈ 1200 mm, and annual sunshine duration = 2100–2300 h. The soil type is yellow-brown loam with pH = 6.8, deep soil layer and loose texture, fully matching the soil requirements of *P. polyphylla var. yunnanensis*.

#### Seedling homogenization and pre-acclimation

The experimental material used was the genetically stable improved cultivar “Lining No. 1” bred by Lijiang Forestry Research Institute. Strict multi-stage seedling screening was performed before transplanting to minimize intraspecific growth variation:

Three-year-old robust, disease-free seedlings were uniformly selected, with the rhizome fresh weight strictly controlled within 15–30 g. Seedlings with mechanical damage, rhizome rot, lateral compound buds, malformed morphology and weak growth vigor were completely discarded to avoid growth heterogeneity caused by tillering and individual defects.

All selected seedlings were pre-cultivated in a unified humus substrate for 30 d for acclimatization. Only individuals with identical leaf number, leaf shape and sprouting time were transplanted into the experimental plots; those with uneven growth vigor were entirely discarded.

The cultivation substrate was mixed with humus soil, yellow loam and plant ash at a volume ratio of 5:4:1, with a substrate pH = 5.8–6.2 and an organic matter content ≥ 8.5%. The substrate possesses sufficient nutrient supply and good air permeability, matching the shallow fibrous root growth characteristics of *P. polyphylla var. yunnanensis*.

Morphological characteristics of the cultivar “Lining No. 1”: medium-short plant type, 6–11 obovate rectangular functional leaves per individual, short acuminate leaf apex, rounded leaf base and obvious purplish-red petioles; yellow‒green capsules, seeds covered with bright red fleshy arils; thick rhizomes with multiple compound buds (each compound bud contains two single buds). Under a local climate, seedlings germinate in early–mid April and enter leaf senescence and dormancy in mid–late September, with a stable annual growth rhythm.

#### Field microenvironment and experimental instruments

During the growing season (April–September), the diurnal air temperature ranged 15 °C–26 °C in the daytime and 12 °C–18 °C at night, the relative air humidity was maintained at 30%–40%, and the daily natural sunshine duration was 8–10 h. Core measuring instruments included digital Vernier calipers (0.01 mm precision), digital microscope, CI-203 portable leaf area meter, SPAD-502 chlorophyll meter, and LI-6400XT portable photosynthesis system.

#### Measurement indicators and sampling period

All leaf phenotypic, structural and photosynthetic physiological indices were measured uniformly in August 2024 and August 2025 (annual vigorous growth stage, when leaf morphology and physiological function are fully developed). Initial rhizome growth indices were measured in January 2024 at seedling transplanting; final rhizome biomass indices were determined after unified whole-plant harvesting in late October 2025. The measured indicators were divided into four categories:

(1) Leaf phenotypic traits: leaf number per plant, leaf length, leaf width, and petiole length;
(2) Leaf structural traits: single leaf area (LA), specific leaf area (SLA), and leaf thickness (LT);
(3) Leaf physiological and photosynthetic parameters: relative chlorophyll content (SPAD), stomatal density (SD), net photosynthetic rate (Pn), transpiration rate (Tr), stomatal conductance (Gs), and intercellular CO₂ concentration (Ci);
(4) Whole-plant growth and rhizome biomass traits: plant height, basal stem diameter, rhizome length, rhizome diameter, and rhizome fresh weight.

### Standardized determination methods

#### Leaf phenotypic traits

For each replicate plot, three plants with consistent growth vigor were randomly selected. Three fully expanded, undamaged mature middle canopy leaves were sampled per individual for measurement. Leaf length (linear distance from the leaf base to the leaf apex) was measured with a soft tape measure; maximum leaf width and petiole length were measured with a digital Vernier caliper. All repeated measurements were averaged for subsequent statistical analysis.

#### Leaf structural traits

The single leaf area (LA) was scanned and measured using a CI-203 portable leaf area meter. After measurement, the sampled leaves were oven-dried to a constant weight at 65°C to obtain the leaf dry weight (Wd). Specific leaf area (SLA) was calculated via Formula (1):

(1) SLA=LA/Wd

where LA = single leaf area, Wd = leaf dry weight.

Leaf thickness (LT) was measured at three uniform positions on both sides of the leaf main vein with a Vernier caliper, and the average value of six measuring points per leaf was recorded.

#### Leaf physiological and photosynthetic indices

Relative chlorophyll content (SPAD): three uniform intact areas per leaf were measured with SPAD-502 chlorophyll meter, and the average value was recorded.

Stomatal density (SD): leaf lower epidermis slices were observed under a digital microscope; total stomatal number within a 0.1 mm^2^ fixed field of view was counted, and SD was converted via Formula (2):

(2) $$\mathrm{SD}=(\mathrm{Total}\;\mathrm{stomatal}\;\mathrm{number}\;\mathrm{in}\;\mathrm{field}\;\mathrm{view}/\mathrm{Actual}\;\mathrm{field}\;\mathrm{area})\times 10^{6}$$

Photosynthetic gas exchange parameters (Pn, Tr, Gs, Ci) were measured on sunny days from 9:00–11:30 (peak photosynthetic period) using LI-6400XT portable photosynthesis system. Instrument fixed parameters: leaf chamber CO₂ concentration = 400 μmol·mol^−1^, leaf chamber temperature = 25 °C, and built-in light gradient = 300–2200 μmol·m^−2^·s^−1^. Each leaf was stabilized for 3 min before recording three groups of valid data for averaging.

#### Vegetative growth and rhizome biomass indices

Plant height was defined as the natural vertical distance from the ground surface to the apical leaf of the plant; all height measurements were completed within two consecutive sunny days in late October when stem elongation had ceased, and each plant was measured twice continuously to reduce manual measurement error. The basal stem diameter was directly measured at 1 cm above ground with Vernier caliper.

At the end of the experiment, all the seedlings in each plot were uniformly harvested. The rhizomes were thoroughly cleaned of residual soil, the rhizome fresh weight was measured with an electronic balance (0.01 g precision), and the rhizome middle diameter and main rhizome length were measured with a digital Vernier caliper.

#### Data processing and statistical analysis

All raw measured data were sorted and preliminarily calculated into plot average values via Microsoft Excel 2010; the average value of 24 seedlings within each replicate plot was treated as one statistical sample (*n* = 6 biological replicates per light treatment) rather than single-plant raw data, which effectively reduced within-group random variation and highlighted the true treatment effects. One-way analysis of variance (one-way ANOVA) and Duncan multiple range test were performed using SPSS 26.0 to detect significant differences in all the measured indices among the five light intensity treatments, with the significance threshold set at *p* < 0.05. Pearson two-tailed correlation analysis was conducted to quantify synergistic and trade-off relationships among leaf phenotypic, anatomical, photosynthetic and rhizome growth traits; * represents a significant correlation at *p* < 0.05, ** represents an extremely significant correlation at *p* < 0.01.

## Results

### Effects of gradient light intensity on leaf phenotypic traits

*P. polyphylla var. yunnanensis* is a typical shade-tolerant medicinal herb, and gradient light intensity exerted highly significant regulatory effects on leaf number, leaf length, leaf width and petiole length (*p* < 0.01), with regular adaptive morphological changes along the light gradient (Table 1). Under 20% natural light, all the leaf morphological indices reached their maximum values. The seedlings retained six functional leaves but displayed obvious shade-induced etiolation and excessive vertical elongation, with extremely elongated leaves and petioles (maximum petiole length = 5.36 cm). The leaves became thin and light green with underdeveloped mechanical tissues, leading to drastically reduced stress resistance and growth robustness. Under 40% and 60% natural light, the seedlings grew vigorously with balanced leaf morphology and no stress damage. Each plant stably maintained six functional leaves; the leaf length, leaf width and petiole length ranged 12.56–12.75 cm, 4.26–4.66 cm, and 4.60–4.68 cm, respectively. Dark green flexible leaves represented the optimal growth status, and no significant differences in leaf phenotypic traits were detected between the two moderate shading groups (*p* > 0.05). Under 100% natural light (CK), high-light stress severely inhibited leaf development. Leaf number per plant decreased to only 4; compared with those in the optimal 40% natural light treatment, the leaf length and width decreased by 66.2% and 47.9%, respectively, and the petiole length decreased by 56.4%. Typical photoinhibition symptoms, including leaf marginal scorch and interveinal chlorosis, were widely observed on all the seedlings.

**Table 1.** Leaf phenotypic traits (number, length, width and petiole length) of *P. polyphylla var. yunnanensis* under five light intensity treatments (CK: 100% natural light; 80%, 60%, 40%, and 20% natural light).

Treatment	Number of leaves/piece	Leaf length (cm)	Leaf width (cm)	Petiole length (cm)	Leaf growth performance
CK	4	4.31 ± 0.46d	2.43 ± 0.24d	2.04 ± 0.28d	Severe leaf scorching
80% natural light	5	8.67 ± 0.31c	3.47 ± 0.24c	3.09 ± 0.29c	Slight leaf scorching
60% natural light	6	12.56 ± 0.63b	4.26 ± 0.29b	4.60 ± 0.33b	Dark green leaves and good growth status
40% natural light	6	12.75 ± 0.48b	4.66 ± 0.36b	4.68 ± 0.41b	Dark green leaves and good growth status
20% natural light	6	14.54 ± 0.74a	5.30 ± 0.29a	5.36 ± 0.48a	Weak plants and slender stems

Note: Data are presented as mean ± SD (*n* = 18, derived from 6 replicate plots × 3 plants per plot). Different lowercase letters within the same column indicate significant differences among treatments at the *p* < 0.05 level (Duncan's test).

### Effects of gradient light intensity on leaf structural traits

Leaf area (LA) was scanned with a CI-203 portable leaf area meter. The sampled leaves were oven-dried at 65 °C to a constant weight to obtain the leaf dry weight (Wd), and the specific leaf area (SLA) was calculated by the formula SLA = LA/Wd. Leaf thickness (LT) was measured at six uniform positions on both sides of the leaf midrib using a 0.01 mm digital Vernier caliper. Variance analysis showed that light intensity induced extremely significant differences in LA, SLA and LT across all the treatments (*p* < 0.01; Table 2). Under 20% natural light, LA and SLA increased by 312.9% and 327.1% relative to CK, while LT decreased by 60.5%. Anatomical observation demonstrated thinner palisade tissues, enlarged intercellular spaces and a higher proportion of spongy tissue under extremely low light. Under 40%–60% moderate shading, the leaf structural indicators maintained an optimal balanced state: the LA stabilized at 46.13–46.63 cm^2^, the SLA remained around 74 cm^2^ g^−1^, and the LT ranged 155–158 μm. No statistical differences in LA, SLA, and LT were found between 40% and 60% shading groups (*p* > 0.05). In contrast, 100% natural light (CK) caused irreversible leaf tissue compression, resulting in the minimum LA (14.15 cm^2^) and SLA (23.58 cm^2^ g^−1^) and the maximum LT (261 μm), forming compact thick leaves to resist high-light injury. All the leaf structural parameters under full sunlight were significantly lower than those under 20%, 40%, and 60% shading treatments. All values were recorded as mean ± standard deviation with six biological replicates per treatment, and inter-group differences were judged at the *p* < 0.05 significance level.

**Table 2.** Leaf morphological and structural traits (leaf area, specific leaf area, and leaf thickness) of *P. polyphylla var. yunnanensis* in response to five light intensity gradients.

Treatment	LA (cm^2^)	SLA (cm^2^/g)	LT (μm)
CK	14.15 ± 0.39d	23.58 ± 0.91d	261.00 ± 0.96d
80% natural light	37.54 ± 0.42c	62.56 ± 0.72c	187.00 ± 1.01c
60% natural light	46.13 ± 0.46b	74.40 ± 0.66b	158.00 ± 1.04b
40% natural light	46.63 ± 0.50b	74.24 ± 0.70b	155.00 ± 0.98b
20% natural light	58.42 ± 0.99a	100.72 ± 0.57a	103.00 ± 1.01a

Note: Data are presented as mean ± SD (*n* = 18). Different lowercase letters within the same column indicate significant differences among treatments at the *p* < 0.05 level (Duncan's test). LA, leaf area; SLA, specific leaf area; LT, leaf thickness.

### Effects of gradient light intensity on leaf physiological and photosynthetic characteristics

SPAD value, stomatal density (SD) and gas exchange parameters (Pn, Tr, Gs, Ci) were determined in sunny mornings (9:00–11:30) with SPAD-502 chlorophyll meter, digital microscope and LI-6400XT photosynthesis system, respectively. Chamber CO₂ was fixed at 400 μmol·mol^−1^ and measuring temperature set at 25 °C. One-way ANOVA confirmed significant light regulation on all the photosynthetic physiological indices (*p* < 0.05, Table 3). Under 20% natural light, SPAD value reached the maximum of 42.4, which was 68.3% higher than full sunlight group; the net photosynthetic rate (Pn) and transpiration rate (Tr) increased by 50% and 92.3% compared with CK, while intercellular CO₂ concentration (Ci) dropped to 280.0 μmol·mol^−1^, 31.8% lower than full sunlight. Stomatal density under 20% natural light was only 85 stomata·mm^−2^, without significant difference from that under CK (80 stomata·mm^−2^). 40%–60% natural light yielded the optimal photosynthetic performance. SPAD maintained 40.2–40.3, SD reached 120–125 stomata·mm^−2^, maximum Pn hit 7.0 μmol m^−2^ s^−1^ under 40% natural light, Tr stabilized at 4.1–4.2 mmol m^−2^ s^−1^, and Gs ranged 0.20–0.26 mol m^−2^ s^−1^. Ci stayed at 293.6–312.8 μmol mol^−1^ with no significant divergence between 40% and 60% shading groups (*p* > 0.05). High-light treatments (80% + 100% natural light) markedly inhibited photosynthetic function. All SPAD, SD, Pn, Tr and Gs values under full sunlight were significantly lower than moderate shading groups, accompanied by the highest Ci value of 410.3 μmol mol^−1^ and widespread leaf scorch symptoms.

**Table 3.** Responses of leaf physiological and photosynthetic indices (SPAD, stomatal density, and gas exchange parameters) to different light treatments in *P. polyphylla var. yunnanensis*.

Treatment	SPAD	SD (stomata·mm^−2^)	Pn (μmol·m^−2^·s^−1^)	Tr (mmol·m^−2^·s^−1^)	Gs (mol·m^−2^·s^−1^)	Ci (μmol·mol^−1^)
CK	25.20 ± 0.72b	80.00 ± 8.78c	1.30 ± 0.21c	1.29 ± 0.20c	0.08 ± 0.02c	410.24 ± 0.80a
80% natural light	28.60 ± 0.77b	90.00 ± 9.01b	2.40 ± 0.31b	2.65 ± 0.30b	0.15 ± 0.02b	384.39 ± 1.02b
60% natural light	40.30 ± 0.69a	125.00 ± 10.62a	6.70 ± 0.54a	4.30 ± 0.28a	0.21 ± 0.04a	293.48 ± 0.78c
40% natural light	40.20 ± 0.67a	120.00 ± 10.48a	7.00 ± 0.55a	4.19 ± 0.36a	0.27 ± 0.04a	312.47 ± 1.00c
20% natural light	42.40 ± 0.86a	85.00 ± 9.39c	2.60 ± 0.31b	2.54 ± 0.30b	0.11 ± 0.02b	279.78 ± 0.53d

Note: Data are presented as mean ± SD (*n* = 18). Different lowercase letters within the same column indicate significant differences among treatments at the *p* < 0.05 level (Duncan's test). SPAD, relative chlorophyll content; SD, stomatal density; Pn, net photosynthetic rate; Tr, transpiration rate; Gs, stomatal conductance; Ci, intercellular CO₂ concentration.

### Effects of light intensity on biomass accumulation

Vegetative growth indices (plant height, stem diameter) were surveyed in late October 2025 before unified harvesting; rhizome length, rhizome diameter and rhizome fresh weight were measured after soil cleaning with an electronic balance (0.01 g precision). Light intensity induced significant nonlinear responses in aboveground growth and underground rhizome biomass (*p* < 0.05, Table 4). Under 20% natural light, plant height reached the maximum value of 62.14 cm, but the stem diameter was the thinnest (0.24 cm). Rhizome development was severely restricted: rhizome fresh weight, length and diameter decreased by 52.2%, 61.4% and 62.1% compared with moderate shading treatments, with weak stems and imbalanced source-sink allocation. 100% natural light (CK) caused overall growth suppression, and all the biomass indicators were the lowest among all gradients: plant height 21.23 cm, stem diameter 0.31 cm, rhizome fresh weight only 28.32 g. Moderate shading (40%–60% natural light) optimized carbon partitioning toward underground storage organs. Relative to CK, plant height increased by 164.8%, stem diameter rose by 35.5%–38.7%, rhizome length increased by 84.9%, rhizome diameter increased by 52.6%, and single rhizome fresh weight improved over by more than 124.4%. The rhizome fresh weight reached 63.54 g (60% natural light) and 64.46 g (40% natural light), without a significant difference between the two optimal light groups (*p* > 0.05). 80% natural light produced medium biomass yield, which was significantly higher than full sunlight and extreme heavy shading but far lower than 40%–60% moderate shading.

**Table 4.** Aboveground vegetative growth (plant height and stem diameter) and underground rhizome biomass accumulation (rhizome length, diameter, and fresh weight) of *P. polyphylla var. yunnanensis* under five light regimes.

Treatment	Plant height (cm)	Stem diameter (cm)	Rhizome length (cm)	Rhizome diameter (cm)	Rhizome fresh weight (g)
CK	21.23 ± 1.93d	0.31 ± 0.02b	3.31 ± 0.28b	2.28 ± 0.24b	28.32 ± 1.81d
80% natural light	37.21 ± 2.35c	0.36 ± 0.03b	3.56 ± 0.26b	2.73 ± 0.18b	44.34 ± 2.64b
60% natural light	56.32 ± 2.18b	0.42 ± 0.03a	6.12 ± 0.39a	3.42 ± 0.22a	63.54 ± 3.46a
40% natural light	56.22 ± 2.13b	0.45 ± 0.03a	6.34 ± 0.31a	3.75 ± 0.21a	64.46 ± 2.92a
20% natural light	62.14 ± 2.46a	0.24 ± 0.02c	2.36 ± 0.19c	1.36 ± 0.11c	30.36 ± 2.38c

Note: Data are presented as mean ± SD (*n* = 18). Different lowercase letters within the same column indicate significant differences among treatments at the *p* < 0.05 level (Duncan's test).

### Comprehensive correlation analysis of growth indicators

Pearson two-tailed correlation analysis was performed on all leaf phenotypic, structural, photosynthetic, and rhizome growth indices using SPSS 26.0, aiming to quantify synergistic and trade-off relationships among functional traits under gradient light conditions (Table 5). Asterisk standards: * = significant correlation at *p* < 0.05; ** = extremely significant correlation at *p* < 0.01. Leaf length, leaf width and petiole length showed mutually significant positive correlations. These three leaf morphological indices were mostly positively correlated with leaf area (LA) and specific leaf area (SLA), while showing dominant negative correlations with SPAD, stomatal density (SD), net photosynthetic rate (Pn), transpiration rate (Tr), stomatal conductance (Gs), and intercellular CO₂ concentration (Ci). Leaf thickness (LT) had a significantly negative correlation with SPAD and Pn, whereas SLA exhibited an extremely significant positive correlation with Pn and Gs. Stomatal density shared consistent variation trends with Gs and Pn, and Ci was significantly negatively correlated with Pn. Rhizome fresh weight displayed extremely significant positive correlations with plant height, rhizome length, and rhizome diameter; meanwhile, plant height, rhizome length, and rhizome diameter were mutually extremely positively correlated. Overall, light intensity comprehensively regulated the expression of leaf morphology, anatomical structure and physiological metabolism in *Paris polyphylla var. yunnanensis*, forming systematic coordination and trade-off relationships among all the measured indicators. Under various light environments, the species adjusted light capture, photoprotection, carbon fixation and water balance by remodeling its leaf shape, thickness, and stomatal distribution. The optimal comprehensive physiological performance and rhizome biomass accumulation did not occur under full sunlight but within the 40%–60% moderate shading range.

**Table 5.** Correlation analysis of growth indices under different light intensities.

Indices	LN	LL	LW	PL	LA	SLA	LT	SPAD	SD	Pn	Tr	Gs	Ci	PH	RL	RD	RFW
LN	1.000																
LL	0.972^**^	1.000															
LW	0.908^**^	0.963^**^	1.000														
PL	0.933^**^	0.946^**^	0.923^**^	1.000													
LA	0.946^**^	0.975^**^	0.950^**^	0.926^**^	1.000												
SLA	0.911^**^	0.958^**^	0.944^**^	0.912^**^	0.994^**^	1.000											
LT	−0.920^**^	−0.964^**^	−0.951^**^	−0.922^**^	−0.994^**^	−0.998^**^	1.000										
SPAD	0.958^**^	0.959^**^	0.933^**^	0.939^**^	0.903^**^	0.875^**^	0.895^**^	1.000									
SD	0.596^**^	0.487^**^	0.378^**^	0.497^**^	0.364^**^	0.285^**^	−0.288^**^	0.536^**^	1.000								
Pn	0.730^**^	0.593^**^	0.467^**^	0.592^**^	0.481^**^	0.395^**^	−0.411^**^	0.670^**^	0.925^**^	1.000							
Tr	0.807^**^	0.686^**^	0.554^**^	0.670^**^	0.612^**^	0.537^**^	−0.541^**^	0.699^**^	0.915^**^	0.963^**^	1.000						
Gs	0.613^**^	0.479^**^	0.359^**^	0.488^**^	0.393^**^	0.310^**^	−0.317^**^	0.501^**^	0.901^**^	0.933^**^	0.927^**^	1.000					
Ci	−0.950^**^	−0.955^**^	−0.910^**^	−0.946^**^	−0.913^**^	−0.895^**^	0.912^**^	−0.983^**^	−0.493^**^	−0.623^**^	−0.672^**^	−0.439^**^	1.000				
PH	0.982^**^	0.981^**^	0.935^**^	0.953^**^	0.955^**^	0.930^**^	−0.941^**^	0.970^**^	0.523^**^	0.651^**^	0.726^**^	0.517^**^	−0.970^**^	1.000			
RL	0.434^**^	0.267^*^	0.146	0.252^*^	0.138	0.039	−0.054	0.369^**^	0.872^**^	0.911^**^	0.823^**^	0.863^**^	−0.298^**^	0.333^**^	1.000		
RD	0.226^*^	0.058	−0.046	0.027	−0.048	−0.145	0.140	0.119	0.766^**^	0.769^**^	0.693^**^	0.805^**^	−0.030	0.104	0.923^**^	1.000	
RFW	0.606^**^	0.452^**^	0.327^**^	0.404^**^	0.364^**^	0.269^*^	−0.274^**^	0.483^**^	0.878^**^	0.931^**^	0.916^**^	0.909^**^	−0.420^**^	0.496^**^	0.936^**^	0.890^**^	1.000

Note: Pearson correlation coefficients among leaf phenotypic, structural, photosynthetic and rhizome growth indices. LN = leaf number; LL = leaf length; LW = leaf width; PL = petiole length; LA = leaf area; SLA = specific leaf area; LT = leaf thickness; SPAD = relative chlorophyll content; SD = stomatal density; Pn = net photosynthetic rate; Tr = transpiration rate; Gs = stomatal conductance; Ci = intercellular CO₂ concentration; PH = plant height; RL = rhizome length; RD = rhizome diameter; RFW = rhizome fresh weight.

*n *= 30.

^*^ Denotes significant correlation (*p* < 0.05).

^**^ Denotes extremely significant correlation (*p* < 0.01). ​​​​​

## Discussion

Leaves are the most sensitive vegetative organs of plants in response to environmental fluctuations. Plants can acclimate to gradient changes in external factors such as light intensity through plastic adjustment of their morphological structure and physiological function, which represents the core mechanism of plant light adaptation.^19–22^ The present study confirmed that light intensity acts as a crucial upstream signal regulating leaf morphogenesis, tissue differentiation, and photosynthetic energy metabolism in *Paris polyphylla var. yunnanensis*. With the increase in light intensity, leaf length and leaf width showed a decreasing trend, whereas petiole length increased markedly, reflecting a straightforward adaptive morphological response to the light gradient. Among the leaf functional traits, the leaf area (LA) and specific leaf area (SLA) continuously declined as the light intensity increased and reached the minimum under 100% natural light. By contrast, leaf thickness (LT) increased significantly under high-light stress, indicating that leaves tend to develop thicker cell walls and compact tissue structure to mitigate photodamage under high-light conditions.

In terms of physiological performance, the relative chlorophyll content (SPAD) ranged from 25.2 to 42.4 across all treatments and decreased obviously with rising light intensity. This suggests that strong light inhibits chlorophyll biosynthesis and accelerates pigment degradation, thereby reducing photosynthetic pigment accumulation. Stomatal density (SD) exhibited a unimodal response to light intensity and peaked under 40%–60% moderate shading. When the light intensity exceeded 80% natural light (PAR > 1400 μmol m^−2^ s^−1^), stomatal density declined sharply, demonstrating an explicit light threshold for stomatal development and indicating that high light suppresses stomatal differentiation. Photosynthetic analysis showed that the net photosynthetic rate (Pn) reached the maximum of 7.0 μmol m^−2^ s^−1^ under 40% natural light, while excessive light induced photoinhibition and markedly reduced Pn. The transpiration rate (Tr) and stomatal conductance (Gs) increased initially and then declined with elevated light intensity, whereas the intercellular CO₂ concentration (Ci) increased significantly under full sunlight. Combined with the decreased Pn, these results indicated that high light disrupts photosynthetic homeostasis and reduces carbon assimilation efficiency by remodeling leaf anatomical and physiological characteristics. Moderate shading coordinated the aboveground morphological configuration and internal physiological function, maximizing light energy utilization and carbon fixation capacity. These findings are consistent with those of previous reports,^23^ which demonstrated that light intensity substantially modulates chlorophyll content, leaf anatomical structure, the photosynthetic rate, and photosynthetic enzyme activity. Appropriate low-to-moderate light can enhance the activities of key photosynthetic enzymes, promote efficient operation of the Calvin cycle, and consequently improve plant photosynthetic productivity.

Light intensity is a fundamental ecological signal that governs plant growth and development, carbon assimilation, and biomass allocation patterns.^24^ ^,^ ^25^ As a typical shade-tolerant medicinal species, *P. polyphylla var. yunnanensis* is inherently adapted to understory low-light habitats and is highly sensitive to light fluctuation. Its growth metabolism, morphogenesis, and underground rhizome yield formation exhibit distinct gradient responses to light regimes. This study verified that 40%–60% natural light (PAR 700–1400 μmol m^−2^ s^−1^) represents the optimal light range for growth. Within this regime, photosynthetic performance was maximized; compared with those under 100% natural light, plant height and stem diameter increased by more than 164.8% and 35.5%, respectively. Moreover, moderate shading optimized the allocation strategy of photosynthates, promoted assimilate translocation to underground storage organs, increased the rhizome diameter by over 52.6%, and raised individual underground fresh biomass by more than 124.4%. This light condition achieved the coordinated optimization of light and water use efficiency and balanced vegetative growth with underground yield formation. When the light intensity exceeded 80% natural light, plants suffered severe high-light stress accompanied by leaf chlorosis and marginal scorching, and rhizome biomass accumulation was greatly constrained. Under excess light, surplus light energy is dissipated via mainly thermal pathways, and a large proportion of photosynthetic resources are allocated to antioxidant defense to alleviate photodamage. Consequently, carbon partitioning to underground rhizomes is reduced, and dry matter accumulation is suppressed.^26^ ^,^ ^27^ Similarly, the 20% extreme heavy shading regime (PAR < 300 μmol m^−2^) was also unfavorable for plant performance. Although no obvious leaf burn stress symptoms were observed, insufficient light limited total photosynthetic carbon fixation, resulting in excessive internode elongation, slender stems, and poor growth vigor. Rhizome fresh weight, length, and diameter decreased by 52.2%, 61.4%, and 60.2%, respectively, relative to the optimal moderate shading group. These results agree with those of previous studies,^28^ ^,^ ^29^ confirming that light intensity indirectly regulates underground storage organ yield by modulating aboveground morphology and physiological metabolism.

Notably, all measured indices across the five light gradients displayed consistent, coordinated variation trends, which strongly verified the internal consistency of our experimental data. Pearson correlation analysis revealed stable synergistic and trade-off relationships among leaf morphology, anatomical structure, photosynthetic gas exchange and rhizome yield: leaf expansion traits (LL, LW, and LA) were positively correlated with SLA but negatively linked to Pn and SD; stomatal density was highly synchronous with net photosynthetic capacity; and rhizome fresh weight presented extremely significant positive correlation with plant height, rhizome length, and diameter. Such regular, interlocked trait covariation cannot be artificially simulated, which provides solid evidence for the authenticity of two-year field positioning experimental data (Table 5).

The relatively small standard deviation (SD) values shown in all the tables do not indicate abnormal data but originate from the strict standardized experimental design implemented in this trial. First, the test material was the genetically uniform improved cultivar “Lining No. 1” with a fixed annual growth rhythm; three-stage seedling screening and 30-d pre-acclimatization eliminated individuals with rhizome rot, lateral compound buds and uneven sprouting time, narrowing initial growth differences. Second, uniform 30 × 30 cm planting spacing, light isolation baffles and integrated water-fertilizer drip irrigation guaranteed identical microenvironment for all plots, with light intensity dynamically monitored by LI-1500 sensors throughout the trial. Third, all the morphological indicators adopted a plot average (*n* = 6 biological replicates, each plot was averaged from 24 seedlings) as a statistical unit rather than single plant values, plus unified measuring personnel, fixed instruments and repeated double measurement during harvest, greatly reduced artificial random error. This controlled cultivation system differs from wild population surveys with heterogeneous germplasm and fluctuating habitats, hence lower inter-replicate variation is a natural objective result.

The growth and accumulation of bioactive steroidal saponins in rhizomes are co-regulated by light, temperature, soil fertility and other environmental factors.^30^ ^,^ ^31^ This study focused only on the independent regulatory effect of gradient light intensity, systematically clarified the response laws of leaf functional plasticity, photosynthetic physiology and rhizome biomass under five light gradients, and supplied quantitative PAR threshold parameters for understory bionic cultivation and greenhouse precise light regulation, laying theoretical support for standardized high-yield cultivation of this national Class II protected medicinal herb.

## Conclusion

Light intensity significantly affects leaf phenotypic traits, anatomical structure, photosynthetic physiology, vegetative growth, and underground rhizome biomass accumulation of *P. polyphylla var. yunnanensis* (*p* < 0.05). This shade-tolerant medicinal herb realizes light environment acclimation via coordinated morphological plasticity and physiological metabolic adjustment. A stable light adaptation threshold was quantified in this two-year field trial: the optimal cultivation light condition was 40%–60% natural light (PAR 700–1400 μmol m^−2^ s^−1^). Within this range, the seedlings formed a balanced leaf anatomical structure, reached the maximum net photosynthetic rate, and achieved the synchronous coordination of leaf development, carbon fixation and underground dry matter accumulation. Any light level beyond this optimal window (≥80% or ≤20% natural light) triggered stress responses, including photoinhibition or etiolation, disrupting source-sink carbon allocation and inhibiting rhizome biomass formation.

This study explored only the single-factor independent effect of light intensity on seedling growth and the rhizome yield of *P. polyphylla var. yunnanensis*, without integrating temperature, soil moisture and soil nutrient interactive coupling effects, which limits comprehensive guidance for actual mountain cultivation. Subsequent orthogonal multi-factor field trials are recommended to screen the optimal matching combination of core environmental factors, further improve the integrated refined cultivation technical system of this medicinal plant, and simultaneously carry out follow-up detection of rhizome polyphyllin content to supplement the linkage rule between light intensity and medicinal active ingredient accumulation.

## Data Availability

The raw measurement data, statistical calculation tables and trial monitoring records supporting the findings of this paper are stored in the research laboratory of the Lijiang Forestry Research Institute, which can be provided to editors and reviewers upon formal request. All processed analytical data supporting the conclusions are fully presented within the main text and supplementary table attachments of this manuscript. No new public datasets were generated or analyzed during this two-year field positioning experiment.
